# Exogenous Spermidine Alleviated Low-Temperature Damage by Affecting Polyamine Metabolism and Antioxidant Levels in Apples

**DOI:** 10.3390/plants13081100

**Published:** 2024-04-15

**Authors:** Meiqi He, Jia Zhou, Deguo Lyu, Gongxun Xu, Sijun Qin

**Affiliations:** 1 Key Laboratory of Fruit Quality Development and Regulation of Liaoning Province, College of Horticulture, Shenyang Agricultural University, Shenyang 110866, China; 2022220307@stu.syau.edu.cn (M.H.); zhoujia20200809@163.com (J.Z.); lvdeguo@syau.edu.cn (D.L.); 2 Research Institute of Pomology, Chinese Academy of Agricultural Sciences, Xingcheng 125100, China

**Keywords:** spermidine, cold resistance, apple, antioxidant enzymes, polyamine metabolism

## Abstract

Low-temperature stress significantly limits the growth, development, and geographical distribution of apple cultivation. Spermidine (Spd), a known plant growth regulator, plays a vital role in the plant’s response to abiotic stress. Yet, the mechanisms by which exogenous Spd enhances cold resistance in apples remain poorly understood. Therefore, the present study analyzed the effects of exogenous Spd on antioxidant enzyme activity, polyamine metabolism, and related gene expression levels of 1-year-old apple branches under low-temperature stress. Treatment with exogenous Spd was found to stabilize branch tissue biofilms and significantly reduce the levels of reactive oxygen species by elevating proline content and boosting the activity of antioxidants such as superoxide dismutase. It also upregulated the activities of arginine decarboxylase, S-adenosylmethionine decarboxylase, and spermidine synthase and the expression levels of *MdADC1*, *MdSAMDC1*, and *MdSPDS1* under low-temperature stress and led to the accumulation of large amounts of Spd and spermine. Moreover, compared with the 2 mmol·L^−1^ Spd treatment, the 1 mmol·L^−1^ Spd treatment increased the expression levels of cold-responsive genes *MdCBF1/2/3*, *MdCOR47*, and *MdKIN1*, significantly. The findings suggest that exogenous Spd can enhance cold resistance in apple branches significantly. This enhancement is achieved by modulating polyamine metabolism and improving antioxidant defense mechanisms, which could be exploited to improve apple cultivation under cold stress conditions.

## 1. Introduction

Mainly distributed in temperate regions, apples (*Malus domestica*) are one of the most widely cultivated fruits. However, apples are vulnerable to varying degrees of low-temperature damage in cold regions at high latitudes. Among these, winter-freezing injury is an important factor affecting the normal growth and development of apples. Dormancy is a key mechanism for apples to resist winter-freezing damage. During endodormancy, apples improve their low-temperature adaptability by adapting to a slow-changing low-temperature environment [1,2]. However, due to climate change, the growing season is prolonged, the vegetative growth of apples is vigorous, new shoots stop growing late, and low-temperature exercise is insufficient. Under extreme weather conditions with rapid cooling, immature branches are vulnerable to freeze-drying and death, which affect reproductive growth [3]. Therefore, it is important to improve the freezing resistance of apples during dormancy.

Polyamines (PAs) are a class of low-molecular-weight aliphatic nitrogen-containing alkali compounds that are widely distributed in plants, mainly putrescine (Put), spermidine (Spd), and spermine (Spm) [4]. The synthesis and catabolism of PAs have been studied extensively. Put is mainly synthesized by ornithine decarboxylase (ODC) and arginine decarboxylase (ADC) in plants and then converted into Spd and Spm by spermidine synthase (SPDS) and spermine synthase (SPMS), respectively. This process decarboxylates S-adenosylmethionine and provides an aminopropyl moiety via catalysis by S-adenosylmethionine decarboxylase (SAMDC). Additionally, polyamine oxidase (PAO) and diamine oxidase (DAO) are implicated in the terminal catabolism of PAs. DAO catalyzes the production of hydrogen peroxide (H_2_O_2_), ammonia, and 4-aminobutyraldehyde. PAO converts Spm to Spd and Spd to Put [5,6]. Previous studies have shown that PAs, as protective substances and signaling molecules, can bind to biological macromolecules, stabilize the structure of cell membranes, maintain the dynamic balance of reactive oxygen species (ROS), promote the synthesis of osmotic regulators such as proline, and thus improve the tolerance of plants to abiotic stresses [7] such as drought [8], salt [9], and low temperature [10]. Wang et al. found that low-temperature stress significantly increased Put content in tobacco, with cold-resistant varieties accumulating more [11]. Amini et al. found that chickpeas with strong cold resistance had more active PA synthesis and metabolism under low-temperature stress; accumulated a large amount of Put, Spd, and Spm; and had higher antioxidant enzyme activity and a more stable photosynthetic pigment content [12]. Zhang et al. found that the application of exogenous PAs could enhance the cold resistance of cucumbers by increasing the activities of antioxidant enzymes, such as superoxide dismutase (SOD) and peroxidase (POD) [13]. Previous studies have shown that Spd is closely related to cold resistance in plants. Cao et al. found that exogenous Spd was beneficial for accumulating gibberellin and ethylene, reducing abscisic acid content, increasing the activities of SOD and POD, and alleviating the damage caused by ROS to kale seed germination under low-temperature stress [14]. Yang et al. found that tomatoes overexpressing *SlSPDS2* reduced cold stress damage by regulating PA metabolism, antioxidants, and osmotic substances [15].

In the initial phase of our study, we discovered that the cold-resistant Hanfu apple variety was able to accumulate significant levels of Spd in one-year-old branches. This finding emerged from transcriptome and metabolome analyses conducted to examine the response of apple branches to low temperatures and freezing injury during their endodormancy [16]. Many studies have also shown that Spd plays an important role in plant responses to low-temperature stress. However, there have been few reports on the effects of exogenous Spd on cold resistance in apples. Therefore, in the present study, changes in PA metabolism, oxidase activity, and related gene expression were analyzed in apple plants under low-temperature stress following exogenous spraying of different concentrations of Spd during endodormancy. It is speculated that the application of exogenous Spd may lead to a positive correlation between PA metabolism and antioxidant levels and, in turn, improve apple cold resistance. The results of the present study could provide a theoretical basis for the practical application of Spd in apple cold-resistant cultivation.

## 2. Results

### 2.1. Effect of Exogenous Spd on Membrane Damage of Apple under Low-Temperature Stress

**Figure 1:**
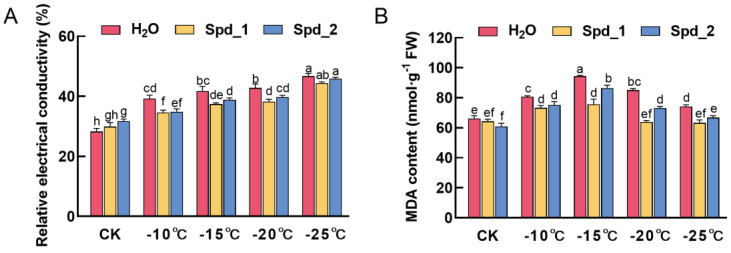
Effects of exogenous Spd on relative electrical conductivity (**A**) and MDA (**B**) content of apple under low-temperature stress. Spd_1:1 mmol·L^−1^ Spd; Spd_2:2 mmol·L^−1^ Spd; control (CK): no low-temperature treatment. Error bars represent mean ± standard deviation (SD) from three replicates. Different letters above the error bar indicate significant differences (*p* < 0.05).

With a gradual decrease in temperature, the relative electrical conductivity value increased slowly and reached a maximum at −25 °C (Figure 1A). Under different low-temperature treatments, exogenous spraying Spd reduced the relative electrical conductivity value; compared with spraying water, the relative electrical conductivity of exogenous spraying 1 mmol·L^−1^ Spd was significantly different (*p* < 0.05). The decrease reached a maximum of −20 °C, a reduction of 10.8%. The MDA content of apples decreased significantly after the exogenous addition of different concentrations of Spd at the same low temperature (*p* < 0.05) (Figure 1B). The treatment effect of 1 mmol·L^−1^ Spd was the best, which decreased by 24.9% at −20 °C, generally greater than other low-temperature treatments. In addition, the MDA content of the water spray treatment at −15 °C was significantly higher than that of other temperatures (*p* < 0.05).

### 2.2. Effects of Exogenous Spd on ROS Metabolism of Apple under Low-Temperature Stress

**Figure 2:**
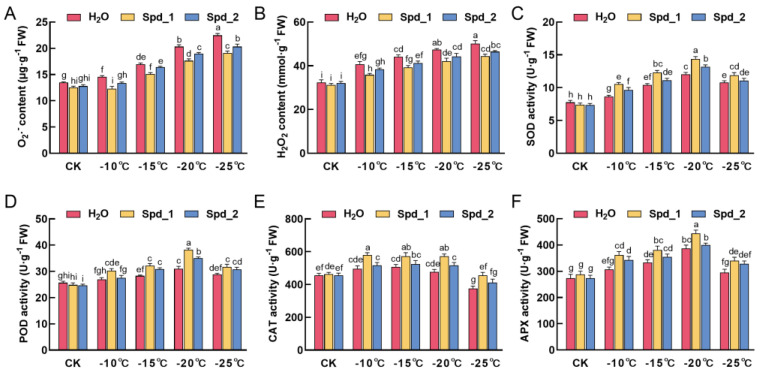
Effects of exogenous Spd on O_2_^.−^ (**A**), H_2_O_2_ (**B**), SOD (**C**), POD (**D**), CAT (**E**), and APX (**F**) of apple under low-temperature stress. Spd_1:1 mmol·L^−1^ Spd; Spd_2:2 mmol·L^−1^ Spd; control (CK): no low-temperature treatment. Error bars represent mean ± standard deviation (SD) from three replicates. Different letters above the error bar indicate significant differences (*p* < 0.05).

Figure 2A,B show that the contents of O_2_^.−^ and H_2_O_2_ increased under low-temperature stress and reached a maximum of −25 °C. Exogenous spraying with different concentrations of Spd reduced ROS content. At −25 °C, 1 mmol·L^−1^ Spd significantly reduced O_2_^.−^ content by 14.9% (*p* < 0.05). At −20 °C, 1 mmol·L^−1^ Spd significantly reduced the content of H_2_O_2_ by 11.2% (*p* < 0.05).

The activity of antioxidant enzymes is closely related to low-temperature tolerance in plants. We observed no significant difference in the effect of different concentrations of Spd on antioxidant enzyme activity in the control (CK), but the activity of antioxidant enzymes increased at low temperatures (Figure 2C–F). Among them, the activities of SOD, POD, and APX reached their maximum at −20 °C, and the activities of SOD, POD, and APX in 1 mmol·L^−1^ Spd were significantly higher than those in 2 mmol·L^−1^ Spd and water, with an increase of 19.6%, 25.1%, and 14.9%, respectively (*p* < 0.05). At −10 °C, −15 °C, and −20 °C, CAT activity changed little and maintained high activity. At −15 °C, the CAT activity of exogenous spraying at 1 mmol·L^−1^ Spd was significantly higher than that of 2 mmol·L^−1^ Spd and water (*p* < 0.05).

### 2.3. Effects of Exogenous Spd on Osmotic Adjustment Substance Content of Apple under Low-Temperature Stress

**Figure 3:**
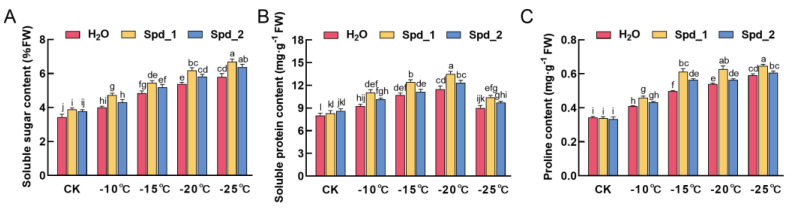
Effects of exogenous Spd on the content of soluble sugar (**A**), soluble protein (**B**), and proline (**C**) in apples under low-temperature stress. Spd_1:1 mmol·L^−1^ Spd; Spd_2:2 mmol·L^−1^ Spd; control (CK): no low-temperature treatment. Error bars represent mean ± standard deviation (SD) from three replicates. Error bars represent the mean ± standard deviation (SD) of three replicates. Different letters above the error bar indicate significant differences (*p* < 0.05).

Figure 3A shows that the soluble sugar content continuously increased and reached a maximum at −25 °C. At −15 °C, −20 °C, and −25 °C, the soluble sugar content of exogenous spraying 1 mmol·L^−1^ Spd and 2 mmol·L^−1^ Spd was not significantly different but higher than that of spraying water (*p* < 0.05). There was no significant difference in soluble protein and proline content between spraying water and spraying different concentrations of Spd in the CK (Figure 3B,C). Under low-temperature treatment, the exogenous addition of different concentrations of Spd further promoted the accumulation of soluble protein and reached a maximum at −20 °C. Among them, 1 mmol·L^−1^ Spd treatment had the best effect, and the increase was obvious at −20 °C, which was 17.7%. At −15 °C, the content of proline in branches increased rapidly under different concentrations of Spd treatment. Among them, the increase was the most significant under 1 mmol·L^−1^ Spd treatment, with an increase of 22.0% (*p* < 0.05). It still maintained a high content of proline at −20 °C and −25 °C. In addition, the soluble protein content in the −20 °C 1 mmol·L^−1^ Spd treatment was significantly higher than that in other temperatures (*p* < 0.05).

### 2.4. Effects of Exogenous Spd on PAs Metabolism in Apple under Low-Temperature Stress

**Figure 4:**
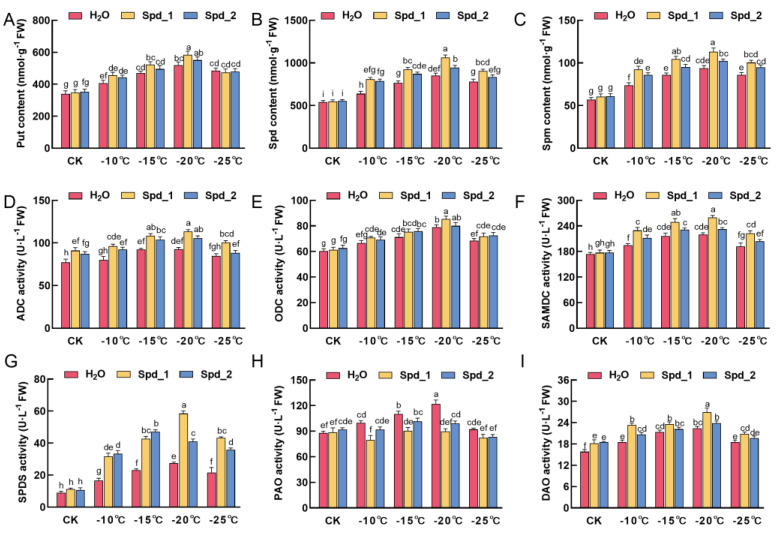
Effects of exogenous Spd on the contents of Put (**A**), Spd (**B**), Spm (**C**), and the activities of ADC (**D**), ODC (**E**), SAMDC (**F**), SPDS (**G**), PAO (**H**), and DAO (**I**) in apple under low-temperature stress. Spd_1:1 mmol·L^−1^ Spd; Spd_2:2 mmol·L^−1^ Spd; control (CK): no low-temperature treatment. Error bars represent mean ± standard deviation (SD) from three replicates. Error bars represent the mean ± standard deviation (SD) of three replicates. Different letters above the error bar indicate significant differences (*p* < 0.05).

Compared to the CK, low-temperature treatment elevated the levels of Put, Spd, and Spm in apple branches, showing an initial increase followed by a decrease as the temperature gradually dropped, with the highest levels observed at −20 °C (Figure 4A–C). Under the same low-temperature treatment, there was no significant difference in the Put content of the branches sprayed with different concentrations of Spd and water. At −10 °C and −15 °C, the Spd content in the branches sprayed with different concentrations of Spd was significantly higher than that sprayed with water (*p* < 0.05). At −15 °C to −25 °C, the Spm content in branches sprayed with 1 mmol·L^−1^ Spd was significantly higher than that sprayed with water (*p* < 0.05), while the Spm content in branches sprayed with 2 mmol·L^−1^ Spd was not significantly different from that sprayed with water.

ADC and ODC are key enzymes in these two synthesis pathways. From Figure 4D, it can be seen that exogenous Spd treatment improved ADC activity. At −10 °C, −15 °C, and −20 °C, the ADC activity of exogenous Spd treatment with different concentrations was significantly higher than that of water spraying (*p* < 0.05). In addition, although the ODC activity of exogenous spraying Spd and water showed an increasing trend at −10 °C, −15 °C, and −20 °C, there was no significant difference in ODC activity under the same low-temperature stress (Figure 4E). SAMDC is a rate-limiting decarboxylase that is involved in the synthesis of Spd and Spm. As shown in Figure 4F, low-temperature stress increased the activity of SAMDC compared with that of CK. Exogenous addition of Spd could further improve the activity of SAMDC at the same low temperature, and the activity of SAMDC sprayed with 1 mmol·L^−1^ Spd was significantly higher than that of 2 mmol·L^−1^ Spd and water at −20 °C (*p* < 0.05). SPDS is a key enzyme involved in Spd synthesis. There was no significant difference in SPDS activity between the different treatments in the CK, but SPDS activity increased in the low-temperature environment. Among them, the SPDS activity of exogenous spraying 1 mmol·L^−1^ Spd at −20 °C was the highest, which was significantly higher than that of exogenous spraying 2 mmol·L^−1^ Spd and water (*p* < 0.05) (Figure 4G). The changes in DAO and PAO activities were relatively stable before and after low-temperature treatment (Figure 4H,I). At −20 °C, the DAO activity of exogenous spraying 1 mmol·L^−1^ Spd was significantly higher than that of spraying 2 mmol·L^−1^ Spd and water (*p* < 0.05), while the PAO activity of exogenous spraying water was significantly higher than that of spraying Spd at −20 °C (*p* < 0.05).

### 2.5. Effects of Exogenous Spd on PAs Metabolism and Cold Resistance Related Gene Expression in Apple under Low-Temperature Stress

**Figure 5:**
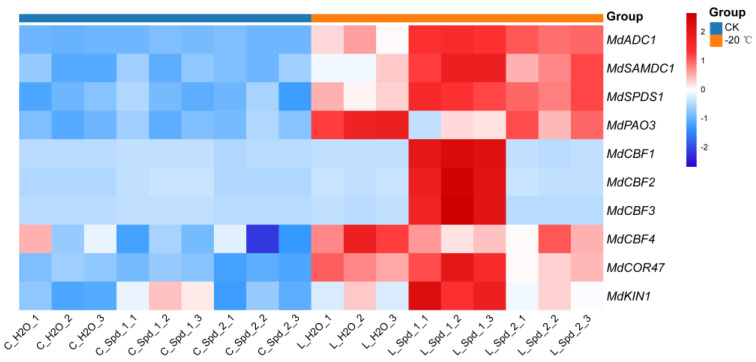
Effects of exogenous Spd on PA metabolism and cold response gene expression in apple under low-temperature stress. Spd_1:1 mmol·L^−1^ Spd; Spd_2:2 mmol·L^−1^ Spd; C: no low-temperature treatment. L: −20 °C.

Based on the physiological indexes of cold resistance, we found that the related indexes changed significantly under the low-temperature treatment of −20 °C. Therefore, we analyzed the expression differences of PA metabolism and cold resistance-related genes in apples under CK and −20 °C. Compared to the CK, a −20 °C low-temperature treatment increased the gene expression of PA metabolism-related enzymes (Figure 5). In addition, compared with exogenous spraying water, exogenous spraying Spd at a low temperature could further increase the relative expression levels of *MdADC1*, *MdSAMDC1*, and *MdSPDS1*. The increase of 1 mmol·L^−1^ Spd was greater, and the expression levels of the three genes increased by 1.7-, 1.8-, and 1.5-fold, respectively. However, the relative expression of *MdPAO3* decreased 0.5-fold. The results reveal that the expression levels of *MdCBF1*, *MdCBF2*, *MdCBF3*, *MdCOR47*, and *MdKIN1* were increased under −20 °C, and the gene expression levels of exogenous spraying at 1 mmol·L^−1^ Spd were higher than those of exogenous spraying at 2 mmol·L ^− 1^ Spd and water (Figure 5).

### 2.6. Correlation Analysis and Principal Component Analysis (PCA)

**Figure 6:**
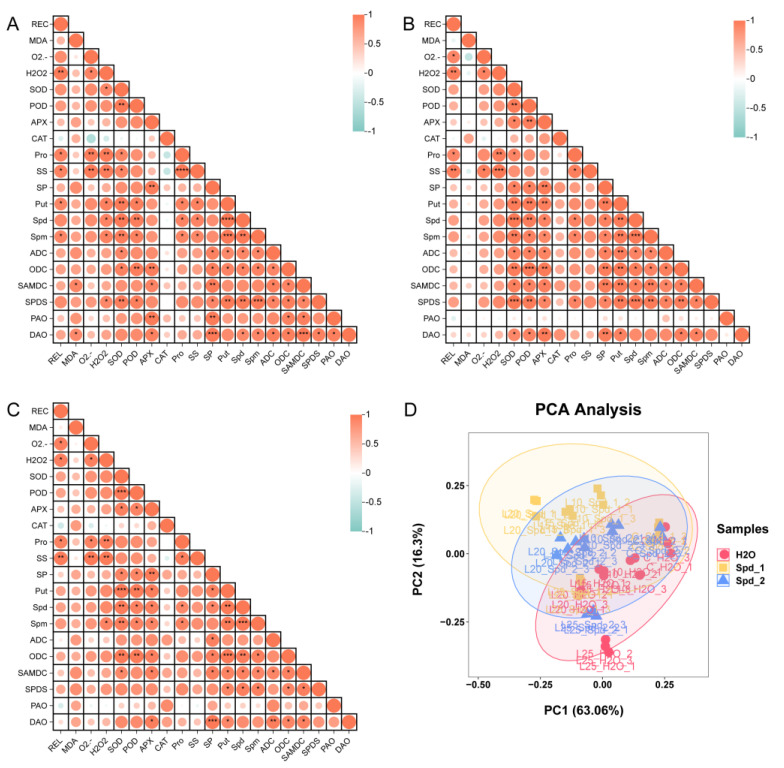
Multivariate analysis of physiological and biochemical indexes. Pearson correlation analysis of the effects of exogenous water spraying (**A**), 1 mmol·L^−1^ Spd (**B**), and 2 mmol·L^−1^ Spd (**C**). PCA of exogenous spraying of different concentrations of Spd (**D**). Relative electrical conductivity (REC); proline (Pro); soluble sugar (SS), soluble protein (SP); Spd_1: 1 mmol·L^−1^ Spd; Spd_2: 2 mmol·L^−1^ Spd. The levels of significance are indicated as follows: * *p* < 0.05; ** *p* < 0.01; *** *p* < 0.001; **** *p* < 0.0001.

The correlations between physiological indexes following the spraying of different concentrations of Spd were analyzed. As shown in Figure 6A–C, Put, Spd, and Spm contents were significantly positively correlated with SOD and POD antioxidant enzyme activities (*p* < 0.05). However, in the 1 mmol·L^−1^ Spd and 2 mmol·L^−1^ Spd treatments, Put, Spd, and Spm contents were significantly positively correlated with APX activity (*p* < 0.05) (Figure 6B,C). In addition, compared with exogenous water spraying and the 2 mmol·L^−1^ Spd treatment, under the 1 mmol·L^−1^ Spd treatment, ADC, ODC, SAMDC, and SPDS were significantly positively correlated with SOD, POD, and APX (*p* < 0.05). SPDS was significantly positively correlated with proline (*p* < 0.05) and extremely significantly positively correlated with Spd (*p* < 0.001) (Figure 6C). In summary, there was a significant positive correlation between PA metabolism and antioxidant levels in the 1 mmol·L^−1^ Spd treatment. The results of PCA showed that the contribution rate of PC1 was 63.06%, and the contribution rate of PC2 was 16.3%. Samples between groups diverged, whereas samples within groups converged, and the repeatability was good (Figure 6D).

## 3. Discussion

Low winter temperatures are important abiotic stress factors that limit the distribution, growth, and development of apples. If low-temperature induction is insufficient or extremely low temperatures occur, apples are prone to freeze injury, causing tree weakness and, in severe cases, death. Therefore, apple production in cold regions should not only select cold-resistant apple varieties but also enhance tree resistance through cultivation regulation. In recent years, salicylic acid [17], methyl jasmonate [18], γ-aminobutyric acid (GABA) [19], and other substances have been reported to improve plant cold resistance.

PAs are plant growth regulators that exist in the form of cations. They can improve the stability of cell membranes by binding to negatively charged membrane phospholipids in cells; affect the biosynthesis of DNA, RNA, and proteins in plants; regulate the process of replication and transcription; and improve the stress resistance of plants by enhancing osmotic adjustment ability and activating antioxidant enzyme systems [20,21]. Therefore, they are widely used in modern agriculture. Previous research found that spraying 0.1 mM Spd on tomato leaves reduces the adverse effects of salt stress on tomato seedlings by increasing antioxidant enzyme activity and proline accumulation [22]. In a study of grape response to drought, Zhao et al. [23] found that exogenous Put could accelerate the ascorbate–glutathione (AsA-GSH) cycle and upregulate the transcription of PA synthesis genes and the accumulation of PAs, thereby improving the drought resistance of grapes. Jalili et al. [24] found that the exogenous application of 5 mM Put could alleviate lipid peroxidation, increase membrane stability in grapes, and reduce frost damage in different grape varieties. However, few studies have investigated the effects of exogenous PAs on cold resistance in apples. Therefore, in this study, we explored the effects of exogenous Spd on cold resistance in apples. These results deepen our understanding of the relationship between the Spd and cold resistance.

Low-temperature stress can cause membrane lipid peroxidation and internal solute extravasation [25]. Relative electrical conductivity and MDA content are important indicators of cell membrane structural damage in plants under low-temperature stress [26]. In this study, the relative electrical conductivity increased with decreasing temperature. However, relative electrical conductivity and MDA content decreased after the exogenous addition of different concentrations of Spd. It was speculated that Spd maintains membrane stability by binding to negatively charged membrane phospholipids, which is similar to the results of previous studies [27,28]. 

Low-temperature stress disrupts the dynamic balance of ROS in plants, leading to oxidative stress and cell death [29]. In this study, the contents of H_2_O_2_ and O_2_^·−^ in one-year-old branches of apple increased under low-temperature stress; however, exogenous spraying of Spd effectively reversed this effect. Nahar et al. found that spraying 0.25 mM Spd on mung bean seedlings could effectively reduce ROS content [30]. Furthermore, previous studies have shown that low concentrations of H_2_O_2_ act as signaling molecules to regulate the low-temperature adaptability of plants [31]. PAs induce antioxidant enzymes to remove excessive ROS [32]. Among them, SOD, as the first line of defense, can undergo a disproportionation reaction to convert O_2_^·−^ into H_2_O_2_, and then POD, CAT, and APX decompose H_2_O_2_ into H_2_O [33]. This study found that exogenous spraying of Spd further increased the activity of antioxidant enzymes under low-temperature stress; exogenous spraying of 1 mmol·L^−1^ Spd exhibited the best results. This has been verified in cucumbers [13], rice [34], centipede grass [35], tomatoes [14], and kale [36]. Therefore, this study shows that exogenous Spd can improve the cold resistance of apples by scavenging ROS. 

Soluble sugars, soluble proteins, and proline can regulate cell osmotic potential, maintain turgor, protect the normal functions of various enzymes and cell membrane structures in plant tissues, and reduce the damage caused by stress [37]. Niu et al. found that the content of osmotic adjustment substances was positively correlated with cold resistance in peaches [38], which is similar to the results of this study. With a decrease in temperature, the content of soluble sugar and proline increased slowly, and the soluble protein peaked at −20 °C. The analysis suggested that large amounts of late embryonic development-rich proteins (LEA) and antifreeze proteins (AFPs) may accumulate. In addition, exogenous Spd increased the content of osmotic adjustment substances. Among them, compared with spraying water and 2 mmol·L^−1^ Spd treatment, exogenous spraying 1 mmol·L^−1^ Spd treatment significantly increased the content of proline (*p* < 0.05). Proline not only maintains the osmotic pressure of cells but also acts as a non-enzymatic antioxidant. Therefore, the accumulation of proline is helpful in improving the tolerance of apples to low-temperature stress. Jankovska-Bortkevic et al. found that the exogenous addition of Spd further promoted the accumulation of proline and improved the survival rate of rape seedlings under low-temperature stress [39], which is similar to the results of this study.

Put, Spd, and Spm are the three most common PAs confirmed to play a crucial role in plant resistance to abiotic stress [40]. Gondor et al. suggested that Spd may play a crucial role in cold acclimation signaling in cereals [41]. We observed that Put, Spd, and Spm increased slowly with decreasing temperature; all peaked at −20 °C. The exogenous spraying of water and Spd had no significant effect on Put (*p* < 0.05). However, exogenous spraying of Spd significantly increased the content of Spd and Spm in one-year-old apple branches (*p* < 0.05). Sheteiwy et al. also found that exogenous Spd positively affected the accumulation of endogenous PAs under low-temperature stress [42]. Spd and Spm contain one and two additionalNH2 groups, respectively, enabling them to perform their protective functions more effectively under stressful conditions [5]. In addition, Spd promotes the accumulation of proline and soluble sugars through signal transduction and activates antioxidant enzymes to reduce the damage caused by low-temperature stress in apples. In addition, although Spd is a naturally occurring compound in plants, it is biodegradable and environmentally friendly [43,44]. However, the cost-effectiveness and eco-friendliness of Spd can be influenced by numerous factors, such as dosage, effectiveness, and environmental impact. Therefore, further research is required to evaluate the economic and environmental benefits of spermidine as an agricultural exogenous application comprehensively.

To elucidate the mechanisms underlying PA fluctuations in apple branches subjected to cold stress, we analyzed changes in PA metabolism-related enzyme activities. In the present study, exogenous spraying of Spd significantly increased ADC activity under low-temperature stress (*p* < 0.05), but there was no significant difference in ODC activity between treatments. It has been speculated that the ADC mainly plays a role in apples under low-temperature stress. Diao et al. found that exogenous application of Spd further increased the ADC activity of tomato seedlings under low-temperature stress [36]. However, Wang et al. suggested that Put synthesis in tobacco seedlings depends on ODC [11]. In addition, exogenous Spd significantly increased the activities of SAMDC and SPDS under low-temperature stress (*p* < 0.05), which may be an important reason for the increase in Spd in one-year-old apple branches. PAO catalyzes the conversion of Spm and Spd to Put. Plants produce H_2_O_2_, GABA, and proline during decomposition. H_2_O_2_ acts as a signaling molecule to activate the antioxidant system. Accumulated GABA and proline can also scavenge excessive ROS and maintain the osmotic balance in cells. In the present study, exogenous spraying of Spd reduced the activity of PAO and increased the activity of DAO under low-temperature stress. Lower PAO activity may be conducive to the accumulation of Spd and Spm, while higher DAO activity is conducive to the accumulation of GABA in one-year-old apple branches, thus improving their cold resistance. Diao et al. found that exogenous Spd increases DAO activity and decreases PAO activity in tomato seedlings under low-temperature stress [36], and Parvin et al. obtained similar results [45]. In addition, combined with correlation analysis, the PA metabolism under stress conditions was positively correlated with antioxidant level, actively maintaining ROS dynamic balance and improving low-temperature adaptability [46].

The activities of key enzymes involved in PA metabolism are closely related to gene expression levels. In the present study, exogenous Spd increased the expression of *MdADC1*, *MdSAMDC1*, and *MdSPDS1* under low-temperature stress. Notably, Zhang et al. overexpressed *CsADC1* in *Arabidopsis thaliana* and observed enhanced cold resistance [47]. Kou et al. confirmed that *SaADC1*-mediated Put synthesis may improve cold resistance in potatoes by enhancing the expression of related genes in the CBF pathway [48]. Jiao et al. found that maize overexpressing SAMDC accumulated large amounts of Spd and Spm and significantly increased the expression of CBF- and cold-responsive genes [49]. Further, tomatoes overexpressing *SlSPDS2* can accumulate proline and soluble sugars at low temperatures and reduce the damage caused by cold stress in tomato seedlings [15]. Zhang et al. reported that *CaSPDS* positively regulates the cold stress response of pepper [50]. Zhang et al. overexpressed *CaPAO2* and *CaPAO4* in Arabidopsis thaliana and found that cold resistance was enhanced in *Arabidopsis thaliana* [51]. In this study, although low temperatures increased *MdPAO3* expression, the exogenous spraying of Spd inhibited the expression of *MdPAO3* compared to the exogenous spraying of water, which was consistent with the change in PAO activity. To further explore the mechanism by which Spd improves cold resistance in apples, we analyzed the changes in the expression of CBF pathway-related genes under low-temperature stress. Among them, the *MdCBF1*, *MdCBF2*, *MdCBF3*, *MdCOR47*, and *MdKIN1* expressions increased and were highest under the treatment of exogenous spraying at 1 mmol·L^−1^ Spd. These results suggest that CBF signaling may play a pivotal role in the regulation of cold resistance via Spd in apples. However, the key genes involved in Spd metabolism and the transcriptional regulation mechanism of the CBF signaling pathway require further investigation.

## 4. Materials and Methods

### 4.1. Plant Materials

The experimental material Hanfu apple (*M. domestica*) was planted at the scientific research base of Shenyang Agricultural University (41°49′ N, 123°34′ E). One-year-old branches of Hanfu apples were collected for sand storage in January 2022. In mid-April 2022, the branches were grafted onto six-year-old Pingyi Tiancha (*M. hupehensis* Rehd. var. pingyiensis Jiang) using the branch grafting method (approximately 60 cm from the ground) and were managed normally during the growing season. That is, watering according to soil moisture conditions, preventing pests and diseases, and regularly mowing weeds in orchards. On 16 September 2022, the whole tree was sprayed with 1 mmol·L^−1^ (Spd_1) and 2 mmol·L^−1^ Spd (Spd_2) every five days, and the CK group was sprayed with the same amount of distilled water. The degree of spraying was based on the fact that the leaves were wet, but there was no droplet condensation or falling, and the treatment was performed four times. Field sampling was conducted on 15 November 2022, and 40 one-year-old branches with good and uniform growth were collected and divided into five groups with eight branches in each group. One group was not treated with low-temperature as the CK, and the other four groups were placed in a low-temperature refrigerator at −10, −15, −20, and −25 °C for one day after reaching the set temperature and then placed in a 4 °C low-temperature refrigerator for one day to thaw. They were then washed with tap and deionized water. After the gauze was wiped dry, one-year-old branches were cut into pieces of approximately 0.5–1 cm by pruning the shears, and the buds were avoided. They were frozen in liquid nitrogen and stored in an ultra-low-temperature refrigerator at −80 °C. Another part of the branch was used to determine the relative electrical conductivity.

### 4.2. Determination of Physiological Indexes Related to Stress Resistance

The relative electrical conductivities of the branches were measured according to the method described by Xu et al. [16]. Malondialdehyde (MDA) content was determined using the thiobarbituric acid method, as described by He et al. [52]. The contents of the superoxide anion (O_2_^.−^) and H_2_O_2_ were determined according to the method of Zhou et al. [53]. SOD, POD, catalase (CAT), and peroxidase (APX) were determined according to the method described by Li et al. [54]. Proline content was determined using the sulfosalicylic acid-acid ninhydrin method, as described by He et al. [55]. The soluble protein content was determined by Coomassie brilliant blue staining according to the method described by Xu et al. [3]. The soluble sugar content was determined by anthrone colorimetry according to the method described by Xu et al. [3].

### 4.3. Determination of Put, Spd, and Spm Content

The PA content was determined by high-performance liquid chromatography (HPLC) according to the method described by Duan et al. [56]. Each branch sample (0.5 g) was ground in 5 mL of 5% (*v*/*v*) perchloric acid. Following a one-hour homogenizing ice bath, the supernatant was collected by centrifugation at 8000 rpm for 30 min at 4 °C. 500 μL of supernatant was added with 1 mL of 2 mol·L^−1^ NaOH and 7 μL of benzoyl chloride, vortexed for 20 s, and reacted at 37 °C for 30 min. A mixture of 2 mL of saturated NaCl solution and 2 mL of diethyl ether was added to extract the benzoyl polyamines. After violent oscillation, the mixture was centrifuged at 5000 rpm for 5 min. The 1 mL diethyl ether phase was collected and dried, and the sample was dissolved with 200 μL methanol before determination. The treated samples were analyzed using a Waters 600 HPLC analyzer. Chromatographic column: Waters reversed-phase C18 column (4.6 mm × 250 mm), column temperature: 30 °C. The eluent was 60% methanol, the flow rate was 0.7 mL·min^−1^, an ultraviolet detector was utilized, the detection wavelength was 230 nm, and the injection volume was 10 μL.

### 4.4. Determination of Key Enzymes in Polyamine Metabolism by ELISA

Samples (0.5 g) were ground with 5 mL of 100 mM phosphate buffer (pH 7.4; TransGen Biotech, Beijing, China), centrifuged at 8000 rpm for 20 min, and the supernatant was collected. The activities of PA metabolism-related enzymes, including ADC, ODC, SPDS, SAMDC, PAO, and DAO, were determined using a Plant ELISA Kit according to the manufacturer’s instructions (Shanghai Enzyme-Linked Biotechnology Co., Ltd., Shanghai, China).

### 4.5. RNA Extraction and qRT-PCR

Total RNA was extracted using a Plant RNAprep Pure Plant Kit (Tiangen, Beijing, China) according to the manufacturer’s protocol. cDNA was synthesized using the TAKARA PrimeScript II 1st Strand cDNA Synthesis Kit (Takara, Dalian, China). Quantitative reverse transcription-PCR (qRT-PCR) was performed on an Applied Biosystems 7500 Real-Time PCR System (Applied Biosystems, Waltham, MA, USA). The actin gene was used as an internal reference gene. Detailed information on the gene primers is provided in Table S1.

### 4.6. Statistical Analysis

Three biological replicates were used for each treatment. The data were analyzed using Statgraphics (STN, St. Louis, MO, USA). All data were tested for normal distribution before analysis. Two-way Analysis of Variance was performed, with Spd concentration and temperature as the main factors. Differences between treatment means were considered significant at *p* ≤ 0.05. Physiological indexes were analyzed by Pearson’s correlation analysis. PCA analysis was performed using the OmicStudio tools at https://www.omicstudio.cn/tool (accessed on 30 March 2024).

## 5. Conclusions

Exogenous Spd spraying can stabilize membrane structure, facilitating the accumulation of osmotic regulators, such as proline, increase antioxidant enzyme activity, and activate PA metabolism. In addition, PA metabolism is significantly positively correlated with antioxidant levels, effectively alleviating oxidative damage caused by low-temperature stress. Compared with the 2 mmol·L^−1^ Spd treatment, the exogenous 1 mmol·L^−1^ Spd treatment increased the expression levels of *MdADC1*, *MdSAMDC1*, *MdCBF1/2/3*, *MdCOR47,* and *MdKIN1* under low-temperature stress significantly. In summary, Spd improved cold resistance in apples by regulating the physiological and biochemical indices of stress resistance and PA metabolism.

## Data Availability

The datasets generated and analyzed during the current study are available from the corresponding author upon reasonable request.

## Supplementary Materials

The following supporting information can be downloaded at https://www.mdpi.com/article/10.3390/plants13081100/s1. Table S1: Primers used for qRT-PCR verification.
